# Supplementary material for: Oral Lactoferrin Supplementation during Induction Chemotherapy Promotes Gut Microbiome Eubiosis in Pediatric Patients with Hematologic Malignancies
Source: Pharmaceutics. 2022 Aug 16;14(8):1705. doi: 10.3390/pharmaceutics14081705 (PMC9416448; doi:10.3390/pharmaceutics14081705)
Supplement: Supplementary file 1 [file pharmaceutics-14-01705-s001.zip › pharmaceutics-1847061-supplementary.pdf]

Article

Oral lactoferrin supplementation during induction chemotherapy promotes gut microbiome eubiosis in pediatric patients with hematologic malignancies

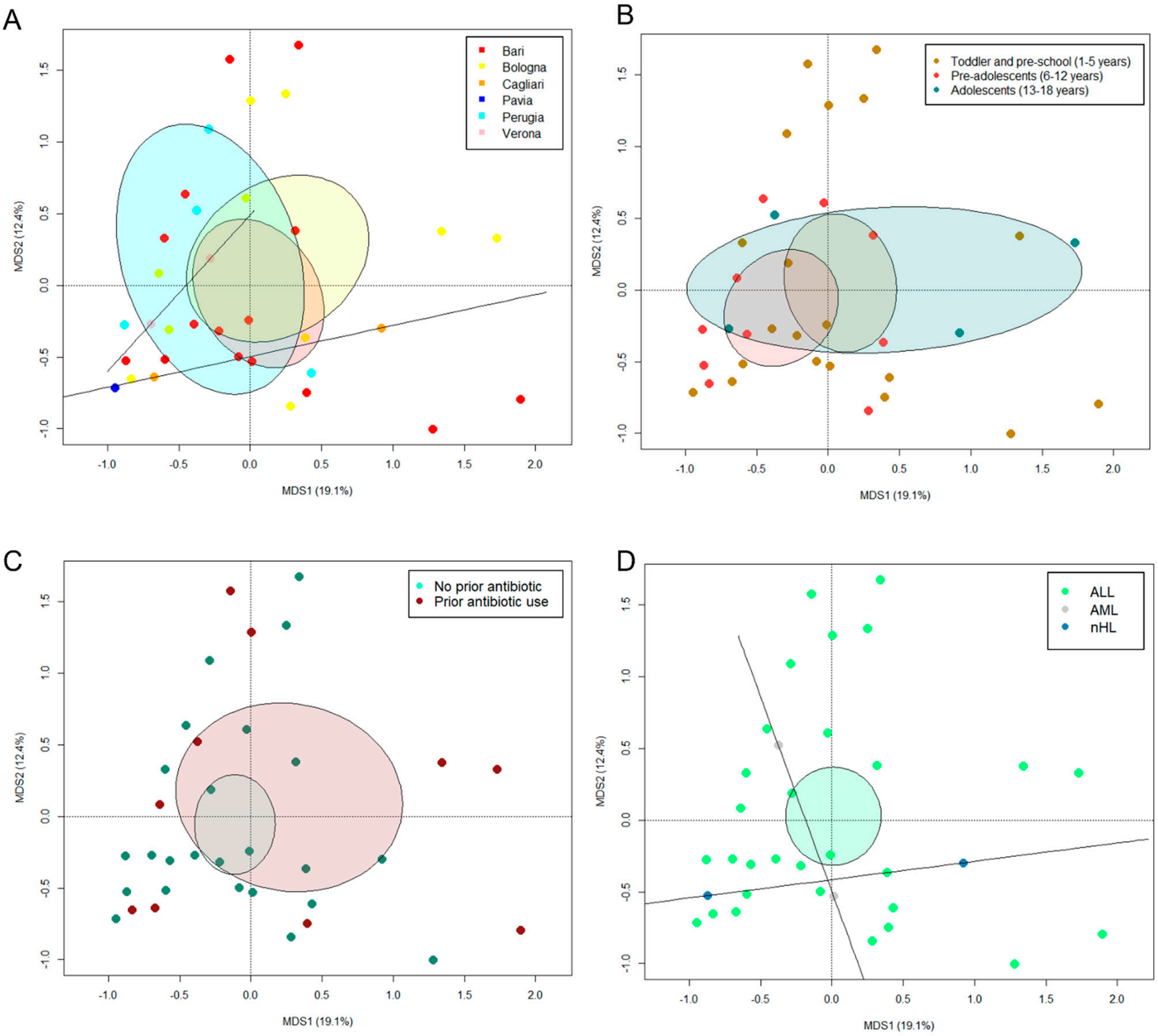

Figure S1. **The baseline gut microbiota of pediatric patients with hematological malignancies does not stratify by recruitment center, age group, prior antibiotic use and disease diagnosis.** PCoA based on Bray–Curtis distances between the gut microbiota profiles of patients before treatment, stratified by recruiting center (**A**, Bari vs. Bologna vs. Cagliari vs. Pavia vs. Perugia vs. Verona), age group (**B**, toddler and preschool vs. pre-adolescents vs. adolescents), prior antibiotic use (**C**, yes vs. no) and disease diagnosis (**D**, ALL vs. AML vs. nHL). Ellipses include 95% confidence area based on the standard error of the weighted average of sample coordinates. No significant separation between groups was found (permutation test with pseudo-F ratio, $p \geq 0.2$).

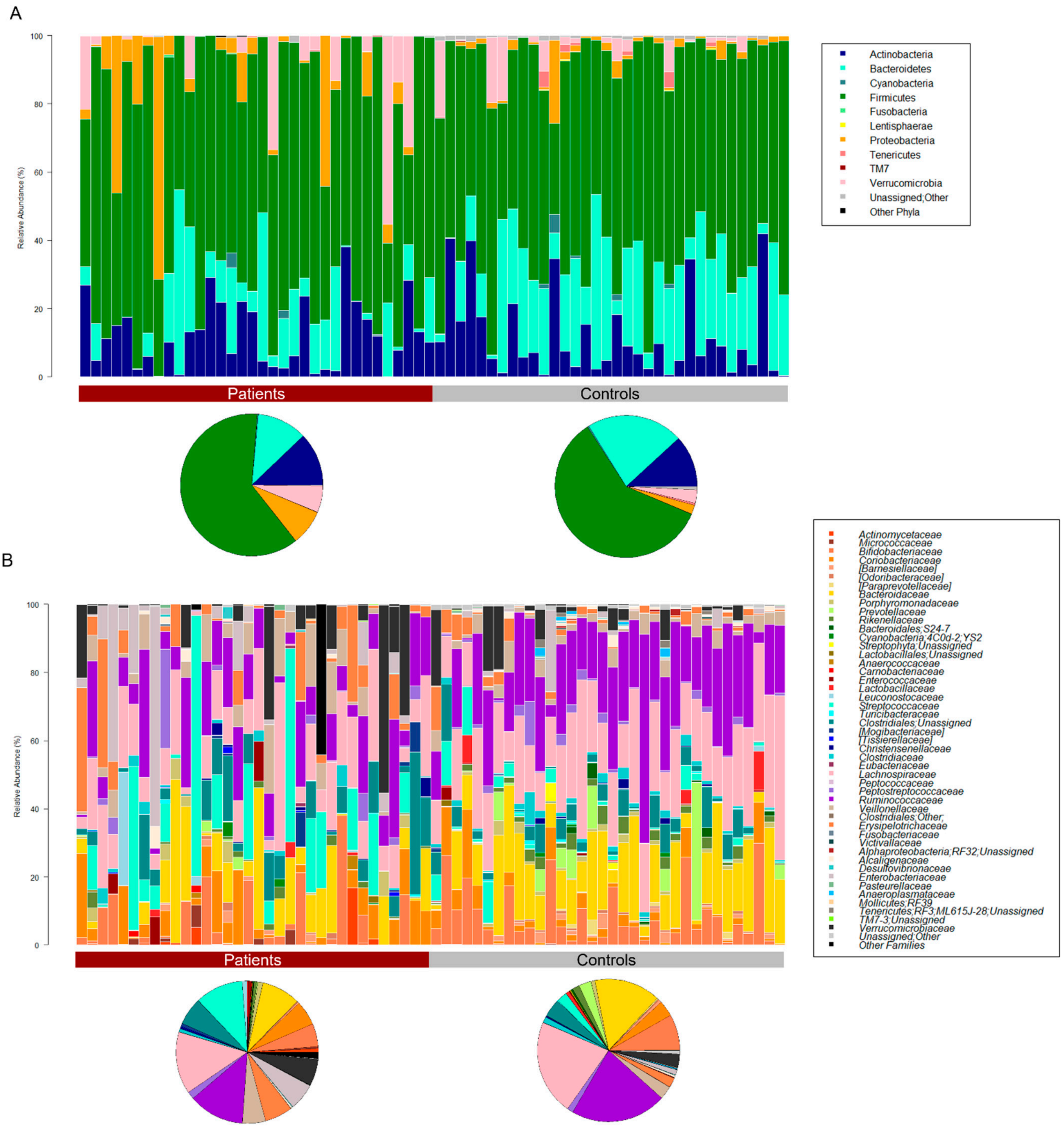

Figure S2. Phylum- and family-level gut microbiota composition of pediatric patients with hematological malignancies compared to healthy controls. On the top of each panel, bar plots showing the relative abundance of major phyla (**A**) and families (**B**) in the gut microbiota of pediatric patients with hematological malignancies vs. age/sex/BMI-matched healthy controls from previous studies [1–3]. On the bottom of each panel, pie charts showing the average relative abundance of major phyla (**A**) and families (**B**) for each study group. Only taxa with relative abundance > 0.1% in at least two samples are shown.

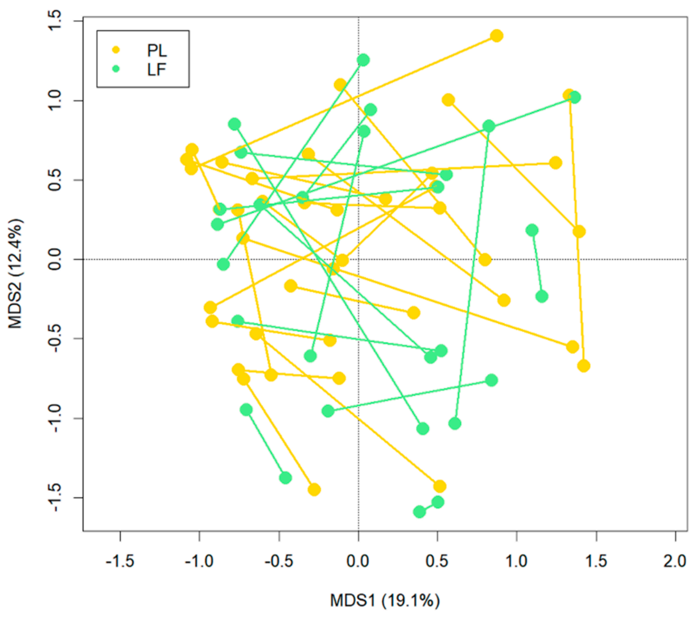

Figure S3. Gut microbiota profiles before and after lactoferrin or placebo supplementation during induction chemotherapy segregate by patient. PCoA based on Bray–Curtis distances between the gut microbiota profiles of patients with hematological malignancies before and after placebo (PL) or lactoferrin (LF) supplementation during induction chemotherapy. Pre- and post-treatment samples from the same subject are connected with a line. A significant separation by subject was found (permutation test with pseudo-F ratio, $p = 0.003$).

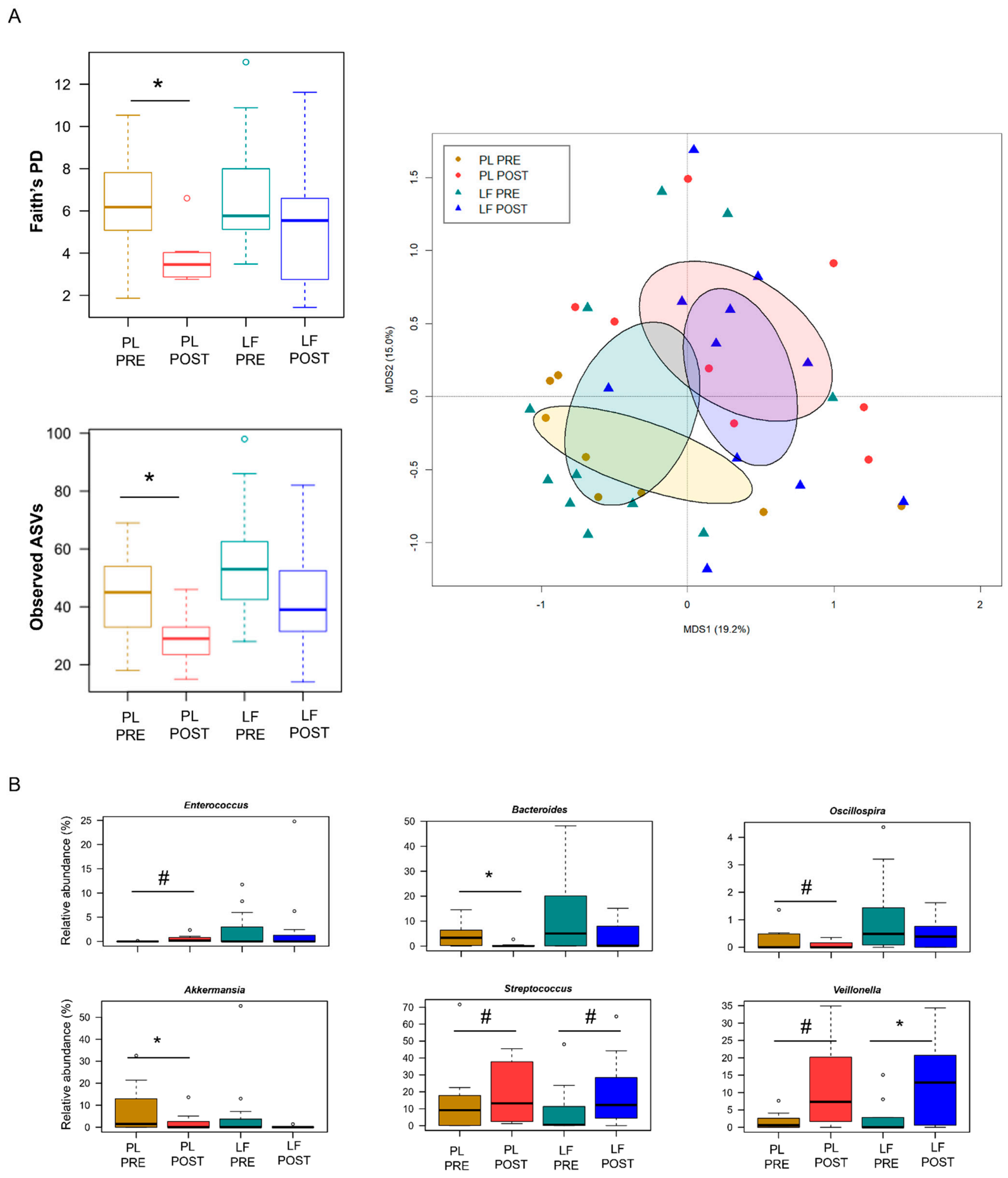

Figure S4. Gut microbiota changes in pediatric oncohematologic patients weighing less than 20 kg, in relation to lactoferrin or placebo supplementation during induction chemotherapy. (**A**) Right, boxplots showing the distribution of alpha diversity, estimated with Faith's phylogenetic diversity (Faith's PD) and the number of observed ASVs, before (PRE) and after (POST) placebo (PL) or lactoferrin (LF) supplementation during induction chemotherapy in patients weighing less than 20 kg and under 6 years of age (Wilcoxon test, * for $p < 0.05$). Left, PCoA based on Bray–Curtis

dissimilarities between the gut microbiota profiles of study subgroups. Ellipses include 95% confidence area based on the standard error of the weighted average of sample coordinates. A significant segregation among groups was found (permutation test with pseudo-F ratio, $p = 0.03$). (**B**) Boxplots showing the relative abundance distribution of genera differentially represented over time in relation to LF or PL supplementation during induction chemotherapy (Wilcoxon test, * for $p \leq 0.05$ and # for $p \leq 0.1$). Only taxa with relative abundance > 0.1% in at least two samples are shown.

References

1. Biagi E, Franceschi C, Rampelli S, Severgnini M, Ostan R, Turroni S et al. Gut Microbiota and Extreme Longevity. Curr Biol 2016; 26: 1480–5.
2. Rampelli S, Guenther K, Turroni S, Wolters M, Veidebaum T, Kourides Y et al. Pre-obese children's dysbiotic gut mi-crobiome and unhealthy diets may predict the development of obesity. Commun Biol 2018; 1: 222.
3. Muleviciene A, D'Amico F, Turroni S, Candela M, Jankauskiene A. Iron deficiency anemia-related gut microbiota dysbiosis in infants and young children: A pilot study. Acta Microbiol Immunol Hung 2018; 65: 551–564.
